# Myosin light chain phosphatase catalytic subunit dephosphorylates cardiac myosin *via* mechanisms dependent and independent of the MYPT regulatory subunits

**DOI:** 10.1016/j.jbc.2022.102296

**Published:** 2022-07-21

**Authors:** Eunyoung Lee, Zhenan Liu, Nhu Nguyen, Angus C. Nairn, Audrey N. Chang

**Affiliations:** 1 Department of Internal Medicine, University of Texas Southwestern Medical Center, Dallas, Texas, USA; 2 Department of Psychiatry, Yale University School of Medicine, New Haven, Connecticut, USA; 3 Pak Center for Mineral Metabolism and Clinical Research, UTSW Medical Center, Dallas, Texas, USA

**Keywords:** cardiac muscle, myosin, protein phosphorylation, protein phosphatase, enzyme, PP1cβ, MYPT1, IEF, isoelectric focusing, MLCK, myosin light chain kinase, MLCP, myosin light chain phosphatase, MYPT, myosin phosphatase target, NA, numerical aperture, RLC, regulatory light chain, TBST, Tris-buffered saline with Tween 20, TCA, trichloroacetic acid

## Abstract

Cardiac muscle myosin regulatory light chain (RLC) is constitutively phosphorylated at ∼0.4 mol phosphate/mol RLC in normal hearts, and phosphorylation is maintained by balanced activities of dedicated cardiac muscle–specific myosin light chain kinase and myosin light chain phosphatase (MLCP). Previously, the identity of the cardiac-MLCP was biochemically shown to be similar to the smooth muscle MLCP, which is a well-characterized trimeric protein comprising the regulatory subunit (MYPT1), catalytic subunit PP1cβ, and accessory subunit M20. In smooth muscles *in vivo*, MYPT1 and PP1cβ co-stabilize each other and are both necessary for normal smooth muscle contractions. In the cardiac muscle, MYPT1 and MYPT2 are both expressed, but contributions to physiological regulation of cardiac myosin dephosphorylation are unclear. We hypothesized that the main catalytic subunit for cardiac-MLCP is PP1cβ, and maintenance of RLC phosphorylation *in vivo* is dependent on regulation by striated muscle–specific MYPT2. Here, we used PP1cβ conditional knockout mice to biochemically define cardiac-MLCP proteins and developed a cardiac myofibrillar phosphatase assay to measure the direct contribution of MYPT-regulated and MYPT-independent phosphatase activities toward phosphorylated cardiac myosin. We report that (1) PP1cβ is the main isoform expressed in the cardiac myocyte, (2) cardiac muscle pathogenesis in PP1cβ knockout animals involve upregulation of total PP1cα in myocytes and non-muscle cells, (3) the stability of cardiac MYPT1 and MYPT2 proteins *in vivo* is not dependent on the PP1cβ expression, and (4) phosphorylated myofibrillar cardiac myosin is dephosphorylated by both myosin-targeted and soluble MYPT-independent PP1cβ activities. These results contribute to our understanding of the cardiac-MLCP *in vivo*.

In cardiac muscle, the physiological role of modulatory phosphorylation of the myosin regulatory light chain (RLC) subunit is distinct from smooth muscles, where contraction/relaxation is tightly regulated by rapid phosphorylation/dephosphorylation of the smooth muscle myosin RLC itself (1, 2). Cardiac muscle RLC is constitutively phosphorylated in beating hearts at around 0.4 mol phosphate/mol RLC and maintained by balanced activities of dedicated cardiac muscle–specific myosin light chain kinase (MLCK) and myosin light chain phosphatase (MLCP). The physiological importance of RLC phosphorylation by the cardiac MLCK has been established by numerous investigators (3, 4, 5, 6). The cardiac MLCK was initially discovered from heart failure patient tissues, as a transcript that is markedly reduced (3). KO mouse studies established that loss of cardiac myosin phosphorylation by the cardiac MLCK caused heart failure (4, 7, 8) and that normal myosin phosphorylation levels were necessary for adaptation to cardiac stress (5). In 2 different models, contrary to hypertrophic phenotype predicted from increased Ca^2+^ sensitivity and increased maximal forces, MLCK overexpression-induced increase in cardiac RLC phosphorylation to 0.6 mol phosphate/mol RLC was associated with protection from maladaptive and physiological hypertrophy (5, 9). These discoveries underscore the importance of maintaining a normal level of cardiac myosin phosphorylation, but information on the phosphatase activity and regulation that balances extents of RLC phosphorylation is lacking.

Understanding of the regulation of MLCP in the heart is largely based on information gained from the study of smooth muscle MLCP, which directly regulate smooth muscle relaxation. The smooth muscle MLCP was defined biochemically as a heterotrimeric holoenzyme comprised of a myosin phosphatase target (MYPT1) regulatory subunit, a PP1cβ catalytic subunit, and a small subunit of unknown function (M20) (10, 11, 12, 13). MYPT1 from smooth and nonmuscle cells targeted PP1cβ to myosin and potentiated the protein phosphatase activity toward phosphorylated myosin (14). Binding of PP1cβ, but not PP1cα or PP1cγ isoforms, to MYPT1 was shown in coexpression studies and detailed in a high-resolution structure (11, 15). MYPT1 protein KO in smooth muscle showed a concomitant reduction in PP1cβ (16, 17) and vice versa (18), consistent with the hypothesis that the interaction of the 2 proteins stabilized the complex *in vivo*. It is not known whether the cardiac MLCP stability *in vivo* is similarly dependent on the coexpression of the regulatory and catalytic subunits.

In the heart, MYPT1 and MYPT2 are both expressed, where they were biochemically shown *in vitro* to bind and potentiate the catalytic activity of PP1cβ toward phosphorylated myosin (Fig. 1) (19). MYPT2 is more abundant than MYPT1, thus thought to be the striated muscle–specific MLCP regulatory subunit. Transgenic overexpression of MYPT2 caused a small decrease in cardiac RLC phosphorylation *in vivo*, presumably due to accumulation of PP1cβ (20). Overexpression of the cardiac muscle–specific small subunit M21 caused cardiac hypertrophy but the mechanisms are unclear (21). MYPT1 has been reported to have potentially important roles in cardiac cell fate through regulation of Nkx2.5 (22) and *in vitro* cell responses to injury through regulation of neurofibromin 2 and YAP (23). It is yet unclear whether *in vivo*, these MYPT1-dependent mechanisms are necessary for cardiac development or function and whether MYPT1 or MYPT2, or both, directly contribute to cardiac MLCP activity.

**Figure 1:**
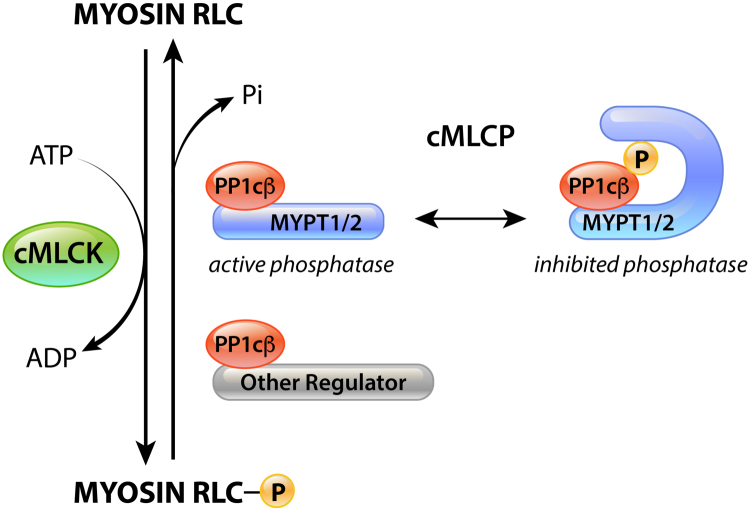
**Schematic of cardiac myosin phosphorylation regulators.** Cardiac myosin is phosphorylated by cardiac myosin light chain kinase (cMLCK). Phosphorylated cardiac myosin is dephosphorylated by the cardiac myosin light chain phosphatase (cMLCP), which is comprised of MYPT1-bound PP1cβ and MYPT2-bound PP1cβ, depicted here as MYPT1/2. The activity of MYPT1/2- bound PP1cβ is regulated by an autoinhibitory intramolecular phosphorylation of MYPT1/2 regulatory subunit. Thus, when MYPT1/2 is phosphorylated, the catalytic activity of PP1cβ is inhibited. PP1cβ is a catalytic subunit that is regulated by other regulators, which may also contribute to dephosphorylation of cardiac myosin, independent of MYPT1/2. MYPT1/2, myosin phosphatase targeting 1/2.

The activity of the MLCP holoenzyme toward phosphorylated myosin is regulated by an intramolecular autoinhibitory phosphorylation of the regulatory subunit MYPT1 (24). The amino acid sequence around the regulatory phosphorylation site Thr696 is conserved between MYPT1 and MYPT2, and the phosphorylation of MYPT1 at this residue was biochemically shown to be sufficient to inhibit the MLCP *in vitro*. *In vivo*, conditional KO studies in smooth muscle have shown that MYPT1 is not necessary for smooth muscle relaxation (17), suggesting other phosphatases could functionally compensate in the absence of the canonical MLCP (10).

Numerous regulatory proteins determine the localization and functional activity of PP1 family of catalytic subunits (1), which are differentially expressed in various organs (25). Characterization of the targeted KO of PP1cβ in cardiac myocytes by others showed compensatory increase in PP1cα protein, PP1cβ KO-specific concentric remodeling, interstitial fibrosis, and contractile dysregulation of the mouse heart (26). These effects specific to the PP1cβ isoform are consistent with an early report that negligible amounts of PP1cα and PP1cγ are expressed in the rat myocardium (25). Cardiac remodeling processes that include marked fibrosis and contractile dysfunction point to among others, activation of inflammatory pathways (27). Enhanced contractility of isolated myocytes and phosphorylation of RLC suggests increased myosin phosphorylation partly contributed to cardiac remodeling and pathology, which were comparable in acute and chronic myocyte-targeted PP1cβ KO models (26). However, interpretation of pathology and insight into mechanisms that link cardiac tissue fibrosis to myocyte-targeted PP1cβ protein reduction is confounded by potential effects on numerous known regulators of PP1 (15). Additionally, although compensatory increase in PP1cα was reported, the localization of the protein was not investigated and effects of PP1cβ protein reduction on the MLCP holoenzyme remains unknown.

Biochemically, from numerous studies of muscle myosin phosphatases, it is understood that purified myosin RLCs are readily dephosphorylated by several different types of protein phosphatases (28, 29, 30, 31). Muscle myosins in its intact form are not promiscuous phosphatase substrates (31), and the necessity of MYPT to target and augment the phosphatase activity toward phosphorylated intact myosin was the dogma until more recent KO studies of the smooth muscle MLCP holoenzyme subunits (17, 18). Extending upon discoveries made in smooth muscles using conditional PP1cβ KO animals (18), we tested the hypothesis that like in smooth muscles, the main catalytic subunit for cardiac MLCP is PP1cβ and that its contribution to maintenance of RLC phosphorylation *in vivo* is dependent on regulation by the striated muscle MYPT2, which targets PP1cβ to the RLC of intact myosin. We used preparative isoelectric focusing (IEF) and optimized separation of proteins by SDS-PAGE to confirm protein levels of PP1c and MYPT isoforms in cardiac muscle extracts and measured the effects of cardiac muscle–targeted PP1cβ KO on other isoforms. Fluorescence immunohistochemistry were used to confirm knockdown of PP1cβ in the cardiac muscle and localize compensatory changes in expression of other isoforms. The effects of PP1cβ protein reduction on extents of cardiac RLC and MYPT phosphorylation and distribution of residual PP1cβ were measured by immunoblots and fractionation studies. A cardiac myofibrillar dephosphorylation assay was developed to measure contributions of endogenous myosin targeted and soluble pools of PP1cβ toward cardiac myosin dephosphorylation. Collectively, the results define biochemical properties of the cardiac muscle MLCP that are distinct from smooth muscle.

## Results

### PP1cβ is the dominant isoform in the heart muscle, and KO causes a compensatory increase in other isoforms

**Figure 2:**
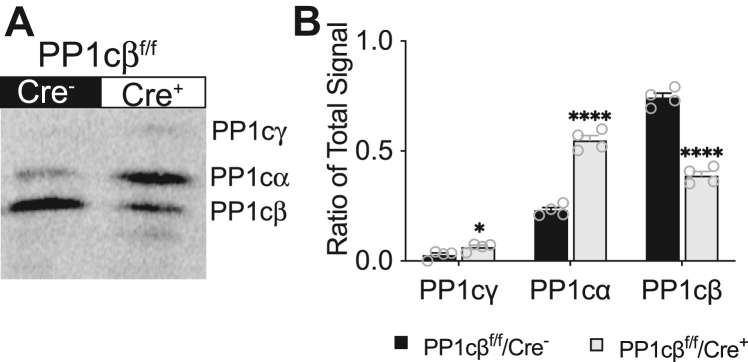
**Quantification of PP1c isoforms in mouse hearts**. *A*, immunoblot of PP1c isoforms using a pan-antibody after separation by preparative IEF. Migration of distinct isoforms is as indicated. Mouse ventricles were dissected and protein extracts from control (PP1cβ^f/f^/Cre^−^) and KO (PP1cβ^f/f^/Cre^+^) animals 2 weeks post-tamoxifen treatment were used. *B*, quantification of indicated isoforms as ratio of total immunoblot signal detected using a pan PP1c antibody in control (PP1cβ^f/f^/Cre^−^, *black bar*) and KO (PP1cβ^f/f^/Cre^+^, *gray bar*). Significance was determined by multiple unpaired *t* tests using GraphPad Prism; ∗*p* < 0.05, ∗∗∗∗*p* < 0.0001.

To measure the ratios of PP1c isoforms expressed in the heart, we used a preparative IEF method that we previously optimized (18), which separates distinct isoforms of PP1c, and detected the expression levels with a pan antibody (Fig. 2*A*). Two weeks post-tamoxifen treatment, relative to total pan PP1c signal per mouse extract, the expression of PP1cγ, PP1cα, and PP1cβ in control animals (PP1cβ^f/f^/Cre^−^) were, respectively, 2.7 ± 1.9%, 23.0 ± 2.5%, and 74.3 ± 4.1%. In the KO animals (PP1cβ^f/f^/Cre^+^), PP1cγ and PP1cα increased by approximately 2-fold to respectively 6.3 ± 1.7% and 54.8 ± 4.5%. The amount of PP1cβ protein was reduced by about half to 38.8 ± 3.7% (Fig. 2*B*).

### PP1cα is increased in myocytes and nonmuscle cells but to a greater extent in myofibroblasts

**Figure 3:**
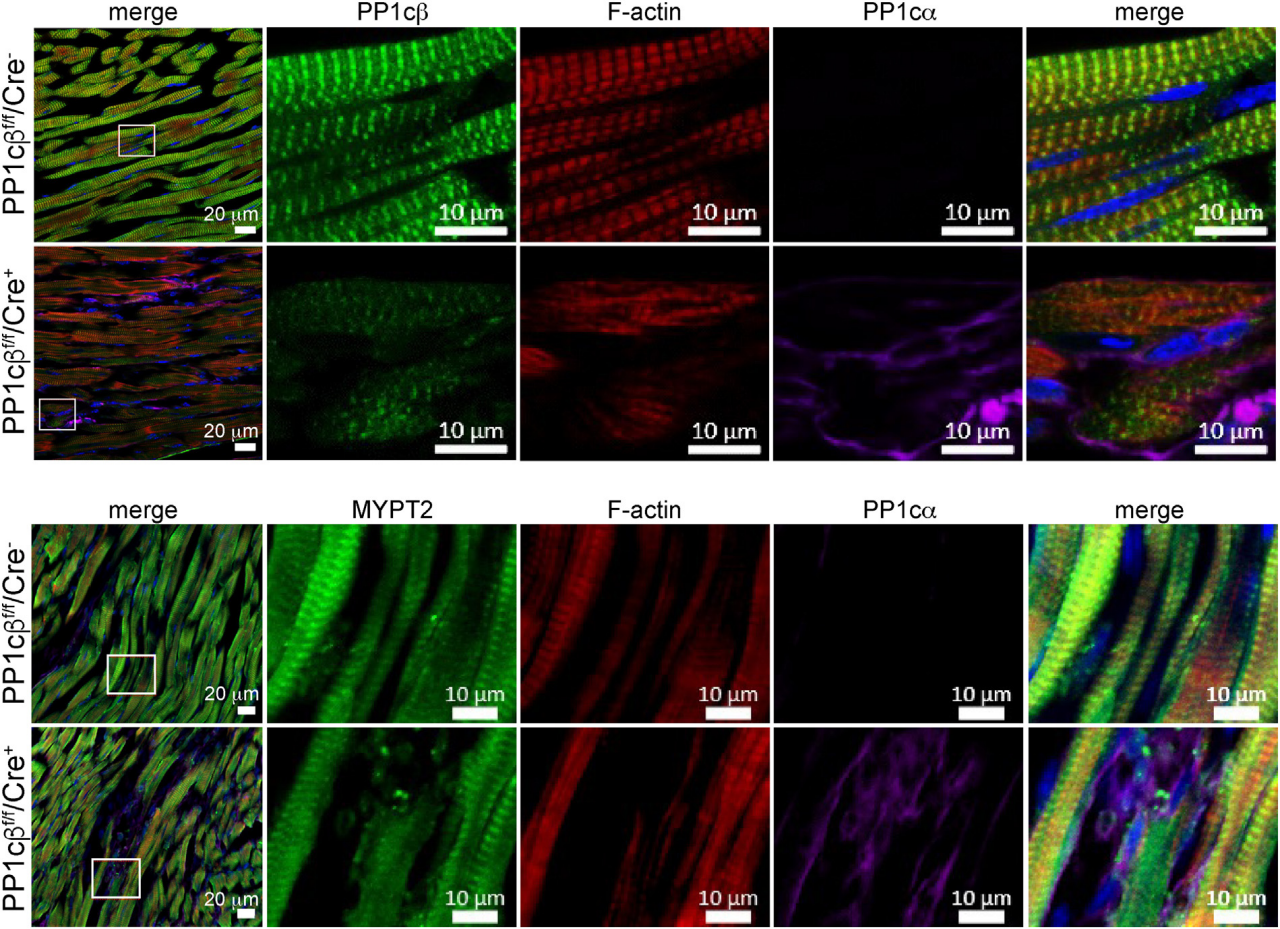
**Localization of PP1cα in control and PP1cβ KO hearts.** Immunofluorescence images of mouse heart sections 2 weeks after tamoxifen treatment to induce KO of PP1cβ. The upper set of 2 rows of panels shows reduced PP1cβ intensity (*green*) and increased PP1cα intensity (*magenta*) in KO heart (PP1c^f/f^/Cre^+^) when compared to control (PP1c^f/f^/Cre^−^). Merged image shows increased PP1cα does not colocalize with PP1cβ. Lower set of 2 rows of panels show no change in MYPT2 intensity (*green*) and increased PP1cα intensity (*magenta*). Merged image shows *magenta* fluorescence appear to be localized to nonmuscle cells. MYPT2, myosin phosphatase targeting 2.

Based on robust compensatory increase in PP1cα protein in PP1cβ KO animals detected by immunoblotting of total heart extracts (Fig. 2), we asked whether PP1cα was upregulated in the same regions of the cardiac muscle where PP1cβ was reduced. The immunofluorescence of PP1cβ was restricted to phalloidin-positive areas. Phalloidin is selective for filamentous actin (F-actin) and in cardiac muscle, stains cardiac actin filaments in the sarcomere. We confirmed reduction of PP1cβ signal in the PP1cβ KO mouse heart by confocal microscopy (Fig. 3, upper panels, *green*), consistent with average total protein reduction measured by immunoblotting (Fig. 2). Coimmunostaining with an antibody against PP1cα (*magenta*) showed PP1cα staining is undetectable in the control tissue under conditions where it is visible in the PP1cβ KO hearts. Increased PP1cα antibody signal was predominantly in areas outside of the sarcomere, where phalloidin-stained F-actin (*red*) was absent. MYPT2, the regulatory subunit of the cardiac muscle MLCP holoenzyme, appeared to be limited to muscle cells of the heart like PP1cβ but were unchanged in the PP1cβ KO (Fig. 3, lower panels).

**Figure 4:**
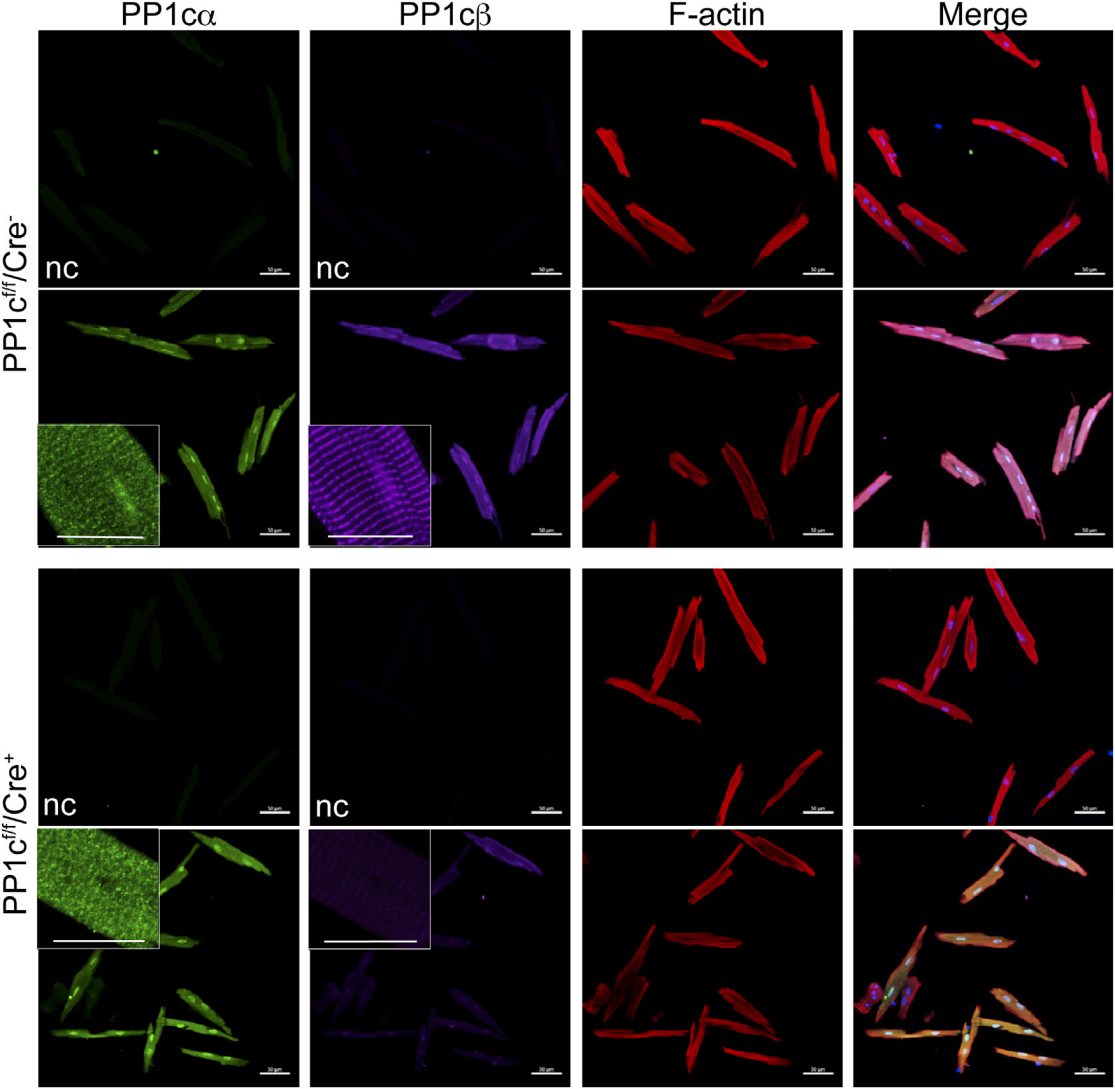
**Immunofluorescence of PP1cα and PP1cβ in isolated cardiac myocytes from control and PP1cβ KO adult mouse.** Cardiac myocytes are isolated from mice that are homozygous for the floxed allele without Cre (PP1c^f/f^/Cre^−^) or from littermates that are homozygous for the floxed allele and Cre-positive (PP1c^f/f^/Cre^+^), 2 weeks after tamoxifen treatment. Myocytes adhered onto laminin-coated coverslips were fixed and stained with antibodies toward PP1cα (*green*) and PP1cβ (*magenta*) as indicated. F-actin was imaged with phalloidin stain and nuclei with DAPI. Cells without primary antibody were imaged with secondary fluorescence antibody in parallel to confirm specificity of the immunofluorescence signal (nc). The scale bar in main panels represents 50 μm. The scale bar in enlarged inset represents 20 μm. DAPI, 4',6-diamidino-2-phenylindole.

To circumvent intense PP1cα antibody signal from nonmuscle cells, which hindered localization of PP1cα within the cardiac myocyte where it is less abundant, we isolated primary adult cardiac myocytes and nonmuscle cells from PP1cβ KO and control animals to separately localize PP1cα, relative to PP1cβ expression. The signal to noise ratio was improved in isolated cardiac myocytes (Fig. 4) and antibody-specific diffuse PP1cα immunofluorescence signal in the cytoplasm and nuclei could be visualized (Fig. 4, *green*). PP1cβ immunofluorescence was similarly present throughout the cell with some localization to the nuclei in the control cells. Higher magnification images showed PP1cβ localized in an even striated pattern consistent with known targeting to muscle myosin by MYPTs (Fig. 4, upper panels, *magenta*). The PP1cβ antibody signal was reduced to nearly undetectable levels in myocytes from KO animals (Fig. 4, lower panels, *magenta*). Robustly increased intensity of PP1cα immunofluorescence in a striated pattern that would indicate compensatory increases at the myofilaments in response to PP1cβ reduction were not observed in the KO myocytes (Fig. 4, lower panels, *green*).

**Figure 5:**
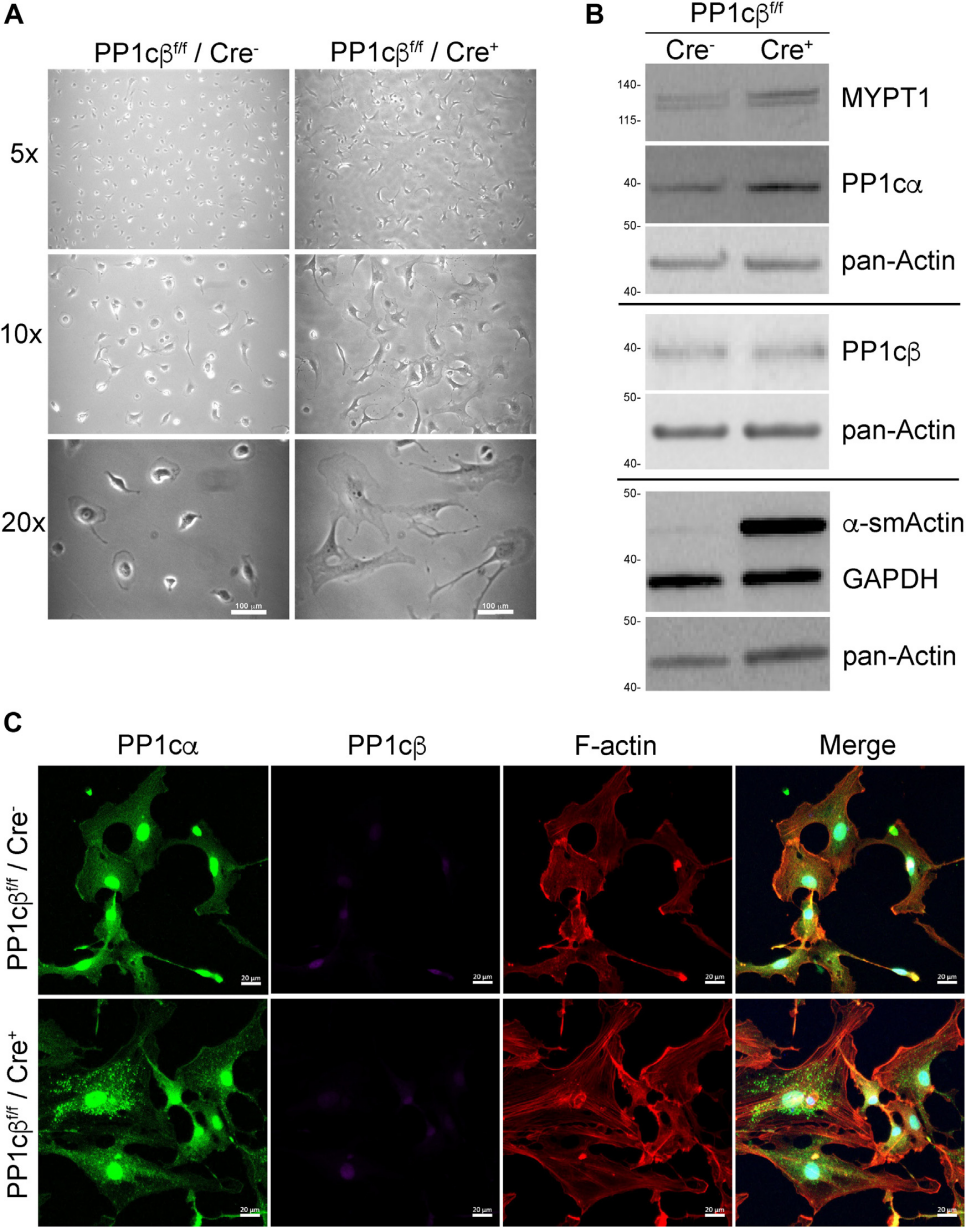
**Cardiac fibroblasts are a source of PP1cα increase in PP1cβ KO hearts.** Equal number of adhesion-enriched fibroblasts from PP1c^f/f^/Cre^−^ control and PP1c^f/f^/Cre^+^ KO hearts 2 weeks after tamoxifen treatment were seeded at <30% confluency and cell morphology imaged after 2 days in culture. *A*, increasing magnification of cells cultured in plastic 6-well dishes show plated cells from KO appear myofibroblast-like, with larger extensions. *B*, representative immunoblots of total lysates from isolated primary adult cardiac fibroblast cells collected after 2 days in culture. Proteins detected are indicated and show increased expressions of MYPT1, PP1cα, and smooth muscle α-actin. *C*, immunofluorescence of isolated adult mouse cardiac fibroblasts from control and KO animals cultured on glass coverslips. Control cells show diffuse PP1cα staining in the cell cytoplasm and increased intensity in the nuclei and edges of the cell periphery. KO cells show PP1cα signal is increased in the cytoplasm where it is distributed in puncta pattern. The PP1cα immunofluorescence in the nucleus is not different between cells from control and KO hearts. PP1cβ immunofluorescence is similarly undetectable in fibroblasts. F-actin staining shows fibroblasts from KO hearts are larger, with greater amount of cytoplasmic stress fibers than control cells, consistent with immunoblots. MYPT1, myosin phosphatase targeting subunit-1.

Adhesion-selected primary fibroblasts from nonmuscle cells of the heart were cultured to determine whether the increase is a result of activated myofibroblasts. After 2 days in culture, the cardiac fibroblasts from the KO animals are more enriched in advanced myofibroblast-like cells, which are partly the source of PP1cα increase, as determined by live-cell morphology, and immunoblots that showed increases in MYPT1, PP1cα, and smooth muscle α-actin protein expression, without changes in PP1cβ (Fig. 5, *A* and *B*). Increase in advanced myofibroblasts from the KO heart is consistent with increased smooth muscle α-actin immunofluorescence (Figs. S1 and S2) and increased cardiac fibrosis reported by others (26). Lack of differences between amounts of PP1cβ in nonmuscle cells isolated from the control and KO animals confirm selectivity of the conditional KO that targeted cardiac myocytes.

Confocal microscopy of adhesion-enriched fibroblasts from control and PP1cβ KO after 3 days of culture on glass coverslips showed cells from the KO were larger, with notably greater amount of cytoplasmic stress fibers visualized by phalloidin staining of F-actin (Fig. 5*C*, *red*). In the control cells, PP1cα antibody distribution was diffuse throughout the cell cytoplasm, with enrichment in the nuclei and at the blebbing edges of the fibroblast membrane (Fig. 5*C*, *green*). PP1cα immunofluorescence in the nonmuscle cells from the PP1cβ KO hearts was brighter in intensity, with pronounced puncta aggregates in the cytoplasm. PP1cβ was essentially undetectable in cells from both WT and KO hearts, consistent with immunoblots of proteins that showed very weak signal for PP1cβ (Fig. 5*B*). Thus, the localization of PP1cα protein in heart sections and distribution of the increased PP1cα protein to nonmuscle cells suggest the increased total expression does not functionally compensate for the reduced PP1cβ in cardiac myocytes.

### Myosin-targeted and soluble PP1cβ are equally reduced with gene ablation

**Figure 6:**
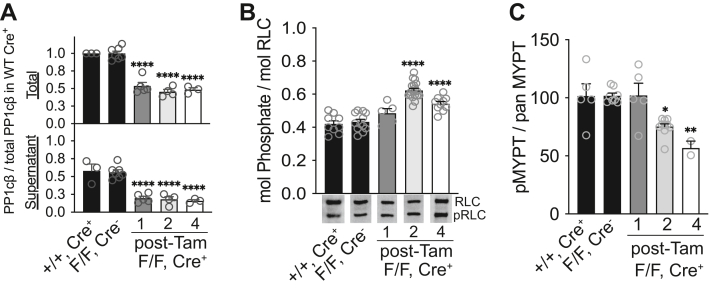
**Time course of PP1cβ gene-ablation effects on distribution of remaining PP1cβ proteins, extents of RLC phosphorylation, and total MYPT phosphorylation.***A*, the amount of PP1cβ protein in the total and supernatant fractions of WT Cre^+^ (+/+, Cre^+^) were compared to PP1cβ^f/f^/Cre^−^ (F/F, Cre^−^) controls and PP1cβ^f/f^/Cre^+^ (F/F, Cre^+^). All mice were treated with tamoxifen. Compared to WT Cre^+^ animals, tamoxifen treatment did not reduce the amount of PP1cβ in homozygous-floxed Cre^−^ animals. Homozygous-floxed Cre^+^ animals had significantly reduced PP1cβ protein in total ventricle homogenates at 1, 2, and 4 weeks after tamoxifen treatment (upper; one-way ANOVA, F(4, 17) = 51.51, *p* < 0.0001). The amount of PP1cβ protein distributed to the supernatant fraction of the ventricle homogenates were similarly reduced compared to controls (lower; one-way ANOVA, F(4, 17) = 24.56, *p* < 0.0001). The amount of PP1cβ in total and supernatant fractions in PP1cβ^f/f^/Cre^+^ groups was all significantly lower than WT Cre^+^. Dunnett’s multiple comparisons post hoc test: ∗∗∗∗*p* < 0.0001. *B*, extents of RLC phosphorylation quantified by immunoblotting denatured proteins after glycerol gel separation of phosphorylated from nonphosphorylated RLC. RLC phosphorylation was significantly increased 2 and 4 weeks after tamoxifen injection (one-way ANOVA, F(4, 49) = 36.36, *p* < 0.0001). Dunnett’s multiple comparisons post hoc test: ∗∗∗∗*p* < 0.0001. *C*, comparison of amount of activated MLCP. Total phosphorylated MYPT protein immunoblot signal was compared to total pan MYPT to quantify effects of tamoxifen-induced PP1cβ protein knockdown at indicated weeks after treatment. Reduction in phosphorylated/total MYPT signal indicates the amount of dephosphorylation-activated MLCP is increased. Phosphorylated/total MYPT levels are significantly reduced by tamoxifen-induced PP1cβ reduction at 2 and 4 weeks (one-way ANOVA, F(4, 25) = 8.158, *p* = 0.0002). Dunnett’s multiple comparisons post hoc test against WT Cre^+^ control: ∗*p* < 0.05, ∗∗*p* < 0.01. MLCP, myosin light chain phosphatase; MYPT, myosin phosphatase target; RLC, regulatory light chain.

In beating mouse hearts that were rapidly removed and snap frozen, MYPT2 is maximally phosphorylated, thus PP1cβ bound to MYPT2 is inhibited (8). In heart muscle homogenates, approximately half of the total PP1cβ cofractionates with MYPT2, which is tightly bound to myosin and insoluble in low ionic strength detergent buffers, and the other half is found in the soluble fraction, presumably bound to other regulators. It is not clear whether both pools of PP1cβ regulate cardiac myosin phosphorylation *in vivo* or if the amount of regulatory subunits is also reduced or activated when PP1cβ protein is reduced. Immunofluorescence of MYPT2 in the mouse hearts suggests the main regulatory subunit of cardiac MLCP is not notably reduced with PP1cβ at 2 weeks after tamoxifen treatment. To test whether differences may have occurred earlier or later than 2 weeks, we compared the extents of RLC phosphorylation and MYPT phosphorylation with the amount of PP1cβ protein in total and supernatant fractions of heart muscle extracts at 1, 2, and 4 weeks post-tamoxifen treatment (Fig. 6). The amount of PP1cβ in the total ventricular muscle extracts reduced significantly and comparably in targeted animals (PP1cβ^f/f^/Cre^+^) at 1, 2, and 4 weeks post-tamoxifen treatment (Fig. 6*A*, upper graph). The amount of PP1cβ distributed to the supernatant fraction in the targeted animals, compared to total amount in WT Cre^+^, were significantly reduced from 56 ± 3.5% in tamoxifen-treated floxed controls (PP1cβ^f/f^/Cre^−^) to 19.8 ± 2.7%, 18.1 ± 3.7%, and 16 ± 1.7%, respectively (Fig. 6*A*, lower graph). These values did not differ from week 1 through week 4. The amount of RLC phosphorylation increased from 0.43 ± 0.01 in controls to 0.48 ± 0.03, 0.62 ± 0.01, and 0.54 ± 0.01 mol phosphate/mol RLC at 1, 2, and 4 weeks post-tamoxifen treatment, respectively (Fig. 6*B*). Thus, at 1, 2, 4 weeks post-tamoxifen injection, PP1cβ was reduced similarly to approximately 50% of the total in controls, and in these samples, a similar ratio of PP1cβ was found in the supernatant fraction where MYPT1 and MYPT2 are not present (8). The total MYPT1/2 phosphorylation was significantly decreased at 2 and 4 weeks post-tamoxifen treatment to 75± 2.4% and 57 ± 6.0% of controls, respectively, suggesting compensatory activation of MLCP in response to reduced total PP1cβ (Fig. 6*C*). Comparison of WT Cre^+^ and PP1cβ^f/f^/Cre^−^ 2 weeks post-tamoxifen treatment showed no measurable differences in the amount of PP1cβ protein distributed to the supernatant fraction (Fig. 6*A*), RLC phosphorylation (Fig. 6*B*), or total MYPT1/2 phosphorylation (Fig. 6*C*). These results show that effects of PP1cβ protein reduction on RLC phosphorylation and MYPT1/2 activation are due to specific loss of the targeted protein and not an artifact of the Cre recombinase transgene.

### Expression of cardiac muscle MYPT1 and MYPT2 are not reduced by cardiac myocyte-targeted PP1cβ gene ablation

**Figure 7:**
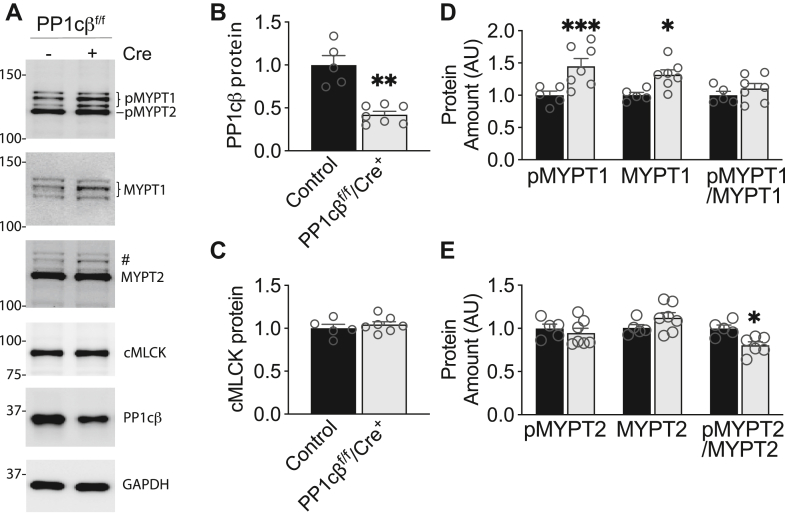
**Effects of PP1cβ KO on expression of MYPT1 and MYPT2.***A*, representative immunoblots of quantified proteins. Floxed animals with and without Cre were sacrificed 2 weeks after tamoxifen treatment. Specific proteins detected are indicated. #The membrane after MYPT1 immunoblot above was reprobed with MYPT2 to compare distinct band below MYPT1. *B*, quantified amount of PP1cβ in control (*black bar*, PP1cβ^f/f^/Cre^−^) and KO (*white bar*, PP1cβ^f/f^/Cre^+^), 2 weeks after tamoxifen treatment. ∗∗*p* < 0.01 by *t* test. *C*, quantified amount of cMLCK. *D*, effects of PP1cβ protein reduction on MYPT1 expression and regulatory phosphorylation. Phosphorylated (pMYPT1) and total (MYPT1) proteins were normalized to Coomassie-stained total extract after separation by 4% to 15% gradient SDS-PAGE. The phosphorylated and total MYPT1 are increased by 45% and 32%, respectively, in the PP1cβ KO heart samples which have about 50% reduction in total protein. ∗∗∗*p* < 0.001, ∗*p* < 0.05 by *t* test. *E*, effects of PP1cβ protein reduction on MYPT2 regulation were measured using same methods as MYPT1. Average phosphorylated (pMYPT2) and total (MYPT2) proteins are not significantly affected by the reduction in PP1cβ protein. Calculated phosphorylated/total MYPT2 shows 20% of MYPT2 is dephosphorylated and activated in the KO samples. ∗*p* < 0.05 by *t* test. cMLCK, cardiac myosin light chain kinase; MYPT1/2, myosin phosphatase targeting 1/2.

Previous work on smooth muscle–specific KO of MYPT1 and PP1cβ has established that the main proteins of the smooth muscle MLCP stabilize each other *in vivo* (17, 18). Selective KO of MYPT1 caused a parallel reduction in PP1cβ and vice versa. Additionally, a compensatory increase in smooth muscle MLCK was measured. We sought to determine whether cardiac muscle MLCP proteins similarly stabilize each other *in vivo* and increase cardiac MLCK expression in response to reduced PP1cβ. At 2 weeks post-tamoxifen treatment, when PP1cβ is reduced to about half of control (Fig. 7, *A* and *B*), the amount of cardiac MLCK was unaffected (Fig. 7, *A* and *C*), and MYPT1 protein in total heart extracts was significantly increased by 32 ± 7.7% (Fig. 7, *A* and *D*). Using a pan antibody, we calculated that the total amount of MYPT1 in WT heart extracts is 30 ± 1.3% and MYPT2 is 69.3 ± 1.1% of the total signal. Thus, increased expression of MYPT1 in response to PP1cβ reduction accounts for approximately 10% of total MYPT. MYPT2 protein expression was not affected by the reduction in PP1cβ (Fig. 7, *A* and *E*), consistent with confocal microscopy images (Fig. 3).

As MYPT-regulated phosphatase activity is itself regulated by an intramolecular autoinhibitory phosphorylation site (Fig. 1) (24), we evaluated the extents of regulatory phosphorylation in the snap frozen total heart extracts from control and PP1cβ KO animals. Total MYPT1 Thr696 phosphorylation was increased by 45 ± 12%, but the ratio of phosphorylated MYPT1 over total MYPT1 was not significantly different due to increased MYPT1 protein expression (Fig. 7, *A* and *D*). The ratio of phosphorylated MYPT2 over total MYPT2 decreased by 20%, indicating 20% of MYPT2 was activated in response to the reduction in PP1cβ (Fig. 7*E*). This value is comparable to total MYPT phosphorylation decrease of 25% at 2 weeks post tamoxifen (Fig. 6*C*).

### Cardiac myofibrillar preparations have active MLCP bound to myosin that balance phosphorylation of myosin *in vitro*

**Figure 8:**
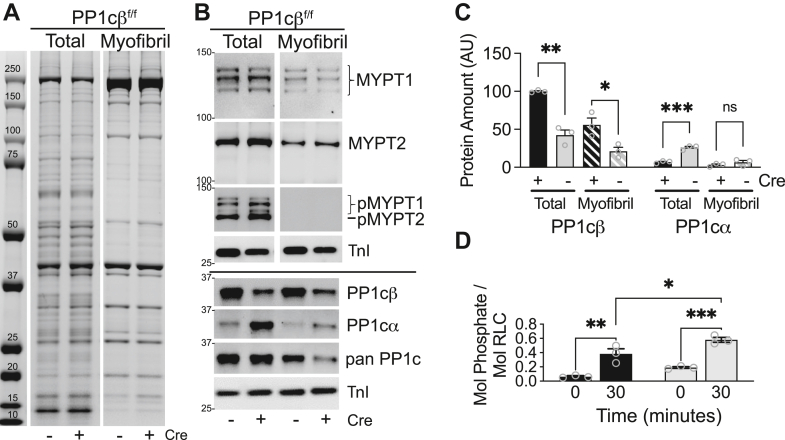
**Effects of PP1cβ KO on myofibrillar MYPT1 and MYPT2.***A*, Coomassie-stained proteins from total heart and myofibrillar protein extracts of control and PP1cβ KO mice hearts. *B*, representative immunoblots of indicated proteins after gel load adjusted by Coomassie-stained myosin heavy chain. Floxed animals with and without Cre as indicated were sacrificed 2 weeks after tamoxifen treatment. Specific proteins detected are indicated. *C*, comparison of total PP1cβ and PP1cα in washed myofibrils relative to control total heart extracts. Significance in difference between control and KO samples were determined by multiple unpaired *t* tests using GraphPad Prism; ∗∗∗*p* < 0.001, ∗∗*p* < 0.01, ∗*p* < 0.05. *D*, extents of myosin RLC phosphorylation before and after 30 min of incubation with Ca^2+^/CaM-dependent MLCK in myofibrils prepared from control (*black bar*, PP1cβ^f/f^/Cre^−^) and KO (*gray bar*, PP1cβ^f/f^/Cre^+^) animals 2 weeks after tamoxifen treatment. The effects of kinase incubation time on RLC phosphorylation were not significantly different between WT and KO (two-way ANOVA, F(1, 8) = 0.738, *p* = 0.415). The extent of RLC phosphorylation after 30 min of kinase incubation was significantly different between WT and KO myofibrils; Sidak’s multiple comparisons post hoc: ∗*p* < 0.05. RLC phosphorylation before (Time = 0) and after (Time =30) kinase incubation was both significantly increased in WT and KO myofibrils; ∗∗*p* < 0.01, ∗∗∗*p* < 0.001. MLCP, myosin light chain phosphatase; MYPT1/2, myosin phosphatase targeting 1/2; RLC, regulatory light chain.

Cardiac myofibrils were prepared from mouse ventricles using procedures previously described (8). Comparison of total heart extracts to washed myofibrils (3 μg each lane) showed enrichment of myofilament proteins in the myofibrils, with no significant differences in Coomassie-stained protein profiles between control and PP1cβ KO samples (Fig. 8*A*). Protein load was adjusted to myosin heavy chain amount determined by Coomassie-stained gel image analysis, and retention of MLCP proteins in washed myofibrils were compared to total heart extract, as shown by representative immunoblot images (Fig. 8). Significant retention of MYPT1 and MYPT2 in the washed myofibrils suggests these proteins are bound to cardiac myosin with high affinity as predicted by the myosin targeting domain sequence (1). Activating dephosphorylation of the MLCP targeted to the myofibrils were confirmed by immunoblotting for MYPT1/2 phosphorylation (Fig. 8*B*). The amount of MYPT1 and MYPT2 proteins in the washed myofibrils were equal (Fig. 8*B*), further confirming that increased MYPT1 signal detected in total heart muscle extracts (Figs. 5*B* and 7*D*) were from nonmuscle cells. Comparison of PP1cβ and PP1cα in the washed myofibrils shows minimal localization of PP1cα to the myofilaments and insignificant difference between the myofibrillar PP1cα from control and PP1cβ KO. Thus, cardiac myofibrils prepared from WT and PP1cβ KO mice have equal amounts of MYPT1 and MYPT2 that are fully activated, but KO myofibrils have half of the amount of PP1cβ compared to total control heart extracts and very low amounts of PP1cα that is similar to WT (Fig. 7*B*).

To interrogate contribution of the myosin-targeted endogenous cardiac-MLCP toward dephosphorylation of cardiac myosin, cardiac myofibrils were phosphorylated by Ca^2+^/CaM-dependent skeletal MLCK using procedures previously described (8). After 30 min of skeletal MLCK and Ca^2+^/CaM incubation in the presence of ATP, the amount of phosphorylated myosin in the KO myofibrils was significantly greater (0.65 ± 0.03 mol phosphate per mol RLC) than in WT myofibrils (0.45 ± 0.01 mol phosphate/mol RLC), suggesting the myofibrillar MLCP is active and contributes to limiting myosin phosphorylation in the myofibrillar preparations (Fig. 7*C*). The extents of myofibrillar myosin phosphorylation *in vitro* under these conditions were similar to endogenous levels measured in snap frozen hearts (Fig. 6*B*).

### Cardiac myofibrillar myosin is dephosphorylated by myosin-bound cardiac MLCP

**Figure 9:**
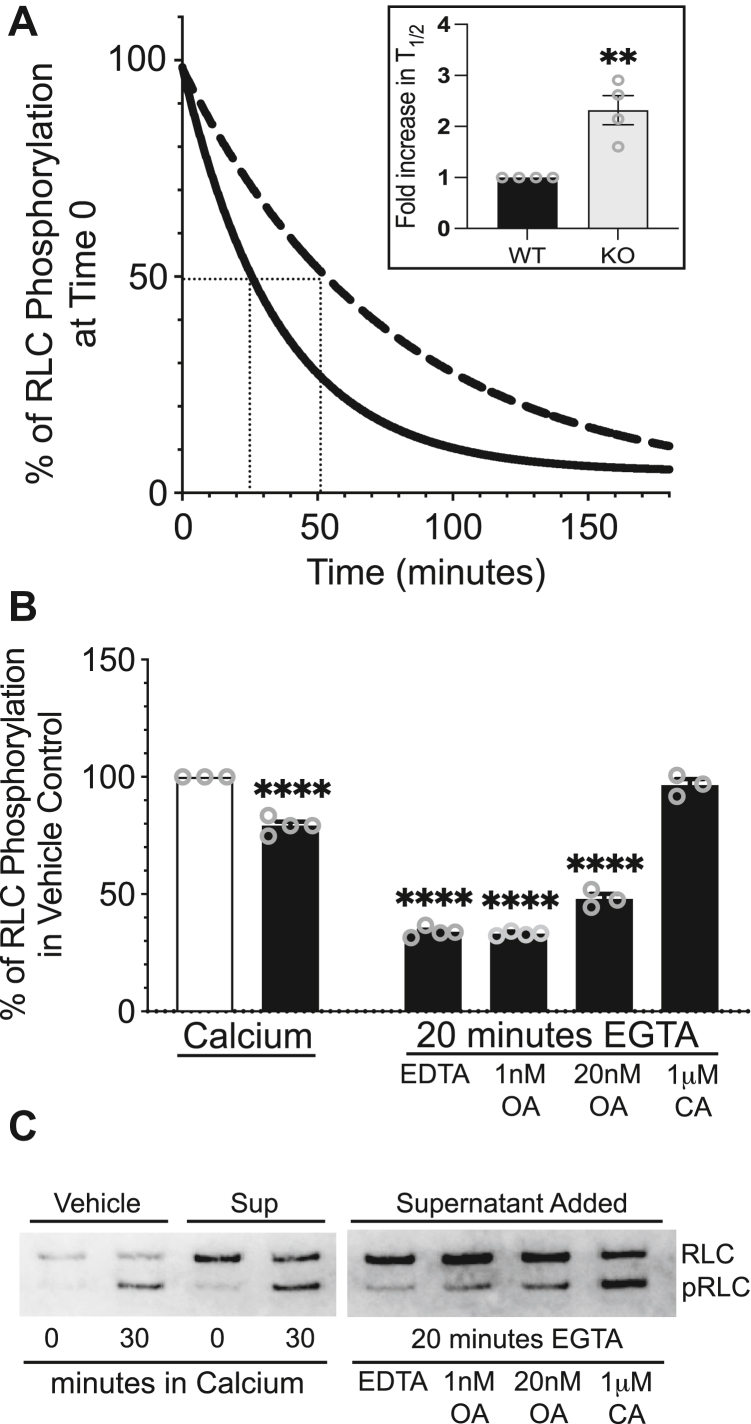
**Dephosphorylation of cardiac myosin in myofibrils.***A*, representative comparison of myosin dephosphorylation by endogenous MLCP retained in myofibrils prepared from control (solid) and PP1cβ KO (dashed) mice hearts. Time to dephosphorylate 50% is shown with *dotted lines*. Boxed inset shows calculated T_1/2_ in KO relative to control WT. Significance was determined by *t* test using GraphPad Prism; ∗∗*p* < 0.01. *B*, dephosphorylation of intact phosphorylated cardiac myosin in myofibrils by added supernatant. Myofibrils were preincubated with buffer (vehicle, *white bar*) or 1:20 dilution of heart extract supernatant fraction (*black bar*) and myosin phosphorylated by skeletal MLCK in the presence of calcium. Dephosphorylation was initiated with the addition of EGTA as shown in panel (*A*). Effects of additional phosphatase inhibitors were compared to confirm phosphatase activity measured with soluble fraction is that of PP1. Significance of differences in RLC phosphorylation in response to added supernatant fraction and inhibitor mixtures were determined by ordinary one-way ANOVA (F(5, 15) = 393.8, *p* < 0.0001). Dunnett’s multiple comparisons post hoc test: ∗∗∗∗*p* < 0.0001. *C*, representative immunoblot of RLC used in quantifications shown in (*B*). MLCK, myosin light chain kinase; MLCP, myosin light chain phosphatase; RLC, regulatory light chain.

Based on evidence of phosphatase activity in the myofibrils that balance the kinase activity *in vitro* (Fig. 8*D*), we compared the relative dephosphorylation rates of myosin in control and PP1cβ KO myofibrils by the myosin-bound MLCP. The extents of myofibrillar myosin phosphorylation were measured after stopping the kinase reaction with EGTA, to ascertain contributions of the myosin-bound MLCP toward RLC dephosphorylation. Average dephosphorylation rate curves show a reduced rate of myosin dephosphorylation in the myofibrils from PP1cβ KO hearts (Fig. 9*A*). Cardiac myosin RLC in myofibrils from PP1cβ KO hearts, which contain about half of the amount of PP1cβ bound to myosin (Fig. 8, *B* and *C*), are dephosphorylated 2-fold slower than WT (Fig. 9*A*, inset).

Based on observations that the MYPT-independent soluble pool of PP1cβ is reduced evenly with the insoluble MYPT-targeted pool in PP1cβ KO hearts (Fig. 4*A*) without changes in the distribution of MYPT protein amounts in the total or supernatant fractions, we asked whether the soluble MYPT-independent phosphatase activity is indeed that of PP1cβ, contrary to long-standing dogma that MYPT is necessary to dephosphorylate intact muscle myosin by PP1cβ. We optimized the supernatant fraction dilution factor that provides measurable myosin phosphatase activity toward myofibrils phosphorylated with MLCK (Fig. S3). When preincubated with a 1:20 ratio of supernatant fraction (*black bars*) or equivalent amount of buffer (*white bar*), the extent of myosin phosphorylation by Ca^2+^/calmodulin-dependent skeletal MLCK was reduced significantly, indicating added phosphatase in the supernatant fraction contributed phosphatase activity to what was already bound to myosin (Fig. 9, *A* and *B*). Known phosphatase inhibitors were used to deduce the identity of the cardiac soluble phosphatase that dephosphorylated myosin after the kinase reaction was stopped with EGTA (32). The main serine/threonine phosphatases in tissue extracts are members of PP1, PP2A, PP2B, or PP2C (33). Complete inhibition of phosphatase activity in the presence of 1 μM calyculin A confirms the catalytic subunit is PP1 or PP2 subtype. The phosphatase activity was not reduced in the presence of additional divalent cation chelator EDTA, indicating the catalytic activity is likely not Ca^2+^-dependent PP2B or Mg^2+^-dependent PP2C. Lack of inhibition of phosphatase activity in the presence of 1 nM okadaic acid indicates the phosphatase is not PP2A, and partial inhibition in the presence of 20 nM okadaic acid confirms biological activity of the okadaic acid used and suggests the soluble phosphatase is similar in activity regulation as the skeletal muscle MLCP (32), which was defined to be PP1. Thus, based on our quantitative IEF of PP1 isoform expression, distribution confirmed by fractionation studies and microscopy and myofibrillar phosphatase activity assays; cardiac myosin is dephosphorylated by both MYPT-targeted PP1cβ as well as PP1cβ bound to a soluble regulator (Fig. 1).

## Discussion

In normal beating hearts, of rodents and humans, cardiac myosin that is phosphorylated at around 0.4 mol phosphate/mol RLC requires constant balancing of kinase and phosphatase activities. We discovered that cardiac myosin is constitutively phosphorylated in beating hearts by Ca^2+^/calmodulin-dependent and Ca^2+^/calmodulin-independent kinase activities (34), but little was known about cardiac myosin dephosphorylation mechanisms that balance out constitutive cardiac MLCK activities. The paucity of information on cardiac myosin dephosphorylation in intact cardiac muscles may be due to inherent difficulties associated with studying a reaction that is not dynamic in normal physiological settings. However, lack of changes in phosphorylation requires dynamic activities of the kinases and phosphatase that are in balance. Human polymorphisms that reduce cardiac myosin phosphorylation and cause dilated cardiomyopathy (35, 36) and *in vivo* studies that show cardiac myosin phosphorylation is necessary for normal cardiac function (4, 5, 6, 8), stresses the need for better understanding of the regulatory mechanisms for cardiac myosin phosphorylation and dephosphorylation.

In an earlier study, we measured that cardiac muscle MYPT2 was maximally phosphorylated at its inhibitory site in snap-frozen tissues that were processed with care to preserve phosphorylations (8). This raised the possibility that like in smooth muscles, another phosphatase regulator was contributing to the dephosphorylation of cardiac myosin to maintain 0.4 mol phosphate/mol RLC. Our biochemical measurements showed that reducing PP1cβ *in vivo* in the heart muscle also caused an increase in baseline myosin phosphorylation to 0.62 mol phosphate/mol RLC. Unlike in smooth muscles, the total amount of MYPT2 and cardiac MLCK were not affected, and since MYPT2 is only found in striated muscle cells, activating dephosphorylation of MYPT2 likely contributed to limiting the extents of cardiac myosin phosphorylation.

Contrary to smooth muscles, where no other PP1c isoforms were differentially expressed in response to the reduction in PP1cβ protein (18), we measured significant increases in PP1cα and MYPT1 over controls in the PP1cβ floxed animal heart extracts 2 weeks after tamoxifen-induced gene ablation from the cardiac myocytes. Consistent with previous characterization of these mice by others, conditional KO of PP1cβ in cardiac myocytes elicited a pronounced fibrosis phenotype, partly attributable to changes in myocyte contractile forces. Various models of heart failure have shown development of cardiac fibrosis in the adult mouse heart could arise from extracellular matrix deposition by various cell types through mechanisms that are not yet clearly understood and are associated with accumulation of nonmuscle cells and myofibroblast-like cells in the myocardium (37). We discovered that activated myofibroblasts and increased macrophages in the heart muscle (Fig. S4) may contribute to increased PP1cα and MYPT1 protein expression measured in total heart extracts. All conventional myosins, including nonmuscle myosin IIA and IIB, are regulated by RLC phosphorylation, which is dephosphorylated by the MLCP. Our results show that in cardiac nonmuscle cells like myofibroblasts and macrophages, MYPT1 and PP1cα are the dominantly expressed MLCP components.

We found that the proportion of PP1cβ distributed to the cytoplasmic fraction of cardiac muscle, where MYPTs are not present, is comparable to the proportion previously measured in smooth muscle (17). Maximal inhibitory phosphorylation of MYPT1 in contracting bladder strips and similarly maximal phosphorylation of MYPT2 in beating hearts (8) indicates that the physiologically active dynamic phosphatase in smooth and striated muscles may be in the cytoplasmic fraction of muscle extracts, comprised of PP1cβ that is regulated by other regulator(s). Indeed, cardiac myosin is rapidly dephosphorylated in total heart homogenates but slowly in noncontracting intact hearts (8). Consistent with this observation, we measured robust phosphatase activity toward phosphorylated myosin in the supernatant fraction of heart extracts.

A caveat to this study and previous measurements of MYPT2 phosphorylation in snap-frozen mouse ventricles is that the mice were under elevated sympathetic tone from the stress of being handled and anesthetized. Thus, inhibitory phosphorylation of MYPTs could be transiently elevated, but effects on cardiac myosin phosphorylation are undetectable due to the low catalytic activity of the cardiac MLCK and slow phosphate turnover rate of cardiac myosin RLC (34, 38). Quantification of proteins relative to total myosin showed, if dephosphorylation of muscle myosins were completely dependent on the MYPT-bound fraction of PP1cβ, there is not enough MYPTs in the muscles to dephosphorylate all myosins (8, 39). The localization of MYPT1 and MYPT2 in relation to the proteins of the thick filament within intact myofibrils needs to be determined to confirm whether MLCP regulation of myosin phosphorylation is constrained to a particular region of the sarcomere. Moreover, we found that although expressed in lesser amount than MYPT2, MYPT1 is present in cardiac myocytes, bound to myofibrillar proteins. The distinct contributions of MYPT1 and MYPT2 toward regulation of cardiac myosin dephosphorylation are unknown and needs to be addressed through gene targeting of MYPT1 and MYPT2 in future studies.

The studies reported herein collectively define MYPT1 and MYPT2 as myofibril-bound regulatory subunits of the MLCP that when activated, dephosphorylates intact myosins in myofibrillar preparations. Distinct from smooth muscles, a reduction in PP1cβ does not cause a parallel decrease in MYPT1 or MYPT2, suggesting these proteins do not costabilize each other *in vivo*. The myofibrillar myosin dephosphorylation rate is augmented by added supernatant fractions of heart homogenates, where the canonical MLCP is not present. Thus, cardiac myosin phosphorylation is regulated by myosin-bound MLCP that is comprised of PP1cβ regulated by MYPT1 and MYPT2, in addition to soluble PP1cβ that is not regulated by an MYPT.

## Experimental procedures

### Ethical approval

Experiments were performed in accordance with the National Institutes of Health and Institutional Animal Care and Use Guidelines. The Institutional Animal Care and Use Committee at the University of Texas Southwestern Medical Center approved all procedures and protocols. Animals were sacrificed by the i.p. administration of a lethal dose of tribromoethanol (250 mg/kg) for tissue collection.

### Generation of genetically modified mice

Mice containing floxed alleles for PP1cβ were crossed with a transgenic mouse line expressing a fusion protein of the Cre recombinase with the modified estrogen receptor-binding domain (CreERT2) under the control of the myosin heavy chain promoter. Mice were bred and screened by Transnetyx. Mice (8–12 weeks old, mixed gender) were injected intraperitoneally with tamoxifen for 5 consecutive days at a dose of 1 mg/day. Hearts were collected on day 14 to 28 as indicated, after start of tamoxifen injection. Mice did not show any visible signs of distress.

### Preparation of total heart extracts

Harvested hearts were removed free of atrium, and ventricles snap frozen within 1 min, using clamps prechilled in liquid nitrogen. Frozen ventricles were ground in liquid nitrogen to uniform heart powder, then stored in aliquots at −80 °C. For immunoblotting, an aliquot of heart powder was simultaneously thawed and precipitated in 10% trichloroacetic acid (TCA) and 10 mM DTT to stop all enzymatic reactions. Precipitated tissue granules were rinsed with ethyl ether (3 times for 10 min each), exposed to air for a few minutes to evaporate the ether, and suspended in at least 30× volume of urea sample buffer containing 8 M urea, 20 mM Tris (pH 8.6), 23 mM glycine, 10 mM DTT, 4 mM EDTA, and 5% sucrose. Proteins were solubilized by extended continuous agitation on a digital Vortex mixer (Ohaus) set at 1400 rpm, with addition of urea crystals to saturation, until samples appeared translucent (6 h at room temperature [RT]). Protein content was determined by Bradford assay (Bio-Rad) with bovine serum albumin as the standard. Samples were aliquoted and stored at −80 °C.

### Separation of PP1 isoforms by preparative IEF

The composition of the preparative IEF minigels was as follows: 9.1 M urea, 5% acrylamide (19:1), 10% glycerol, 2% ampholyte (pH 5–7, SERVA), 2% CHAPS. Gel mixture was degassed for 10 min at RT and polymerized for 1 h after the addition of 0.05% ammonium persulfate and 0.1% *N*, *N*, *N*', *N*' - tetramethylethylenediamine. The gel running buffers were 50 mM L-histidine (inner buffer) and 20 mM phosphoric acid (outer buffer). Frozen heart powder aliquots prepared as described previously were weighed and homogenized in 50× volume of Bio-Rad rehydration solution using a glass homogenizer. Additional urea crystals were added to saturation during continuous shaking at RT for at least 30 min to solubilize heart muscle proteins. The solubilized heart proteins were then clarified by centrifugation at 20K×*g* for 2 min. Following gel polymerization and assembly of the apparatus, wells were prefilled with 25% Bio-Rad rehydration solution, and samples were underlayed to prevent contact with inner buffer. Samples were focused at 400V overnight (∼16 h). Following IEF, gels were removed, washed in 100 ml total of gel fixation buffer (50% methanol and 2% SDS) for 1 h (20 min × 3 buffer changes), washed in transfer buffer for 5 min, and transferred to nitrocellulose membrane in standard wet transfer buffer with 0.1% SDS for 3 h at 10V (Mini Blot transfer apparatus, Thermo, plate electrodes). Transferred proteins on membrane were immunoblotted using procedures detailed below under as “Immunoblotting and antibodies.”

To quantify PP1c isoforms after separation by preparative IEF, distinct protein band intensity in acquired blot images were quantified using Image Lab software (Bio-Rad). Location of specific isoforms after preparative IEF was confirmed previously (18). After image background adjustment, the individual band volumes and total lane volumes from the image analysis software were used to calculate the amount of distinct isoforms as a ratio of total PP1 immunoblot signal. Calculated ratios from four different control and KO mice were graphed using GraphPad Prism (GraphPad Software).

### Fractionation of heart extracts

Mouse ventricles snap frozen using clamps prechilled in liquid nitrogen were pulverized to frozen heart powder in liquid nitrogen, aliquoted, and then stored in vented microfuge tubes in −80 °C until use. Frozen heart powder was homogenized in 30× weight adjusted volumes of heart extraction buffer (50 mM Mops pH 7.4, 2 mM EDTA, 2 mM EGTA, 150 mM NaCl, 1× Halt protease inhibitor cocktail, 10 μM E-64, 1 mM DTT, 1% NP-40) with a ground glass homogenizer incubated on ice for 30 min. An aliquot of total homogenate was removed, then the rest precleared by centrifugation (10,000×*g*, 5 min), prior to separating the supernatant fraction by ultracentrifugation (100k×*g*, 30 min at 4 °C). Equivalent volumes of total and supernatant fractions were denatured in lithium dodecyl sulfate sample buffer (ThermoFisher) following manufacturer instructions, prior to separation by SDS-PAGE.

### Immunoblotting and antibodies

Tissue extract proteins solubilized in urea sample buffer were quantified by Bradford assay (Bio-Rad) and then added to 0.25 volumes of 4× lithium dodecyl sulfate sample buffer and reducing agent for SDS-PAGE per reagent instructions (Thermofisher). Protein solubilization quality and gel loading for immunoblots were predetermined by Coomassie-stained (Imperial Protein Stain, ThermoFisher) gel image analysis after separation by 8% to 15% SDS-PAGE (Bolt, Mops buffer system, ThermoFisher). Proteins (3 μg/lane) were separated by 8% to 15% SDS-PAGE and were transferred onto a nitrocellulose membrane, then processed for immunoblotting using standard procedures (40). Briefly, membranes were incubated with specific antibodies diluted in 5% bovine serum albumin/Tris-buffered saline with Tween 20 (TBST) overnight at 4 °C, washed in TBST 3 × 10 min, probed with horseradish peroxidase–conjugated secondary antibodies at RT for 1 h, washed, then developed using ECL Plus (ThermoFisher), and imaged using Chemidoc MP (Bio-Rad). Antibodies used were as follows: phosphorylated MYPT1 (p696) from Millipore, pan MYPT from Abcam (#32519), and MYPT1 from Cell Signaling (2634s). Pan PP1c and PP1cβ antibodies were described previously (18).

### Measurement of RLC phosphorylation

About 8 M urea sample buffer was subjected to urea/glycerol-PAGE to separate nonphosphorylated from monophosphorylated RLC. Following electrophoresis, proteins were transferred to polyvinylidene difluoride membranes, fixed in 0.4% glutaraldehyde/PBS for 5 min, washed in PBS 3 × 5 min, and probed with a mouse mAb against cardiac myosin (Enzo). The ratio of monophosphorylated RLC to total RLC (nonphosphorylated plus monophosphorylated) was determined by quantitative densitometry of developed immunoblots and expressed as mole phosphate per mole protein.

### Confocal microscopy

Mouse hearts were retrograde perfusion fixed through the aorta in 10% neutral buffered formalin, cryopreserved in 18% sucrose, and embedded in Tissue-Tek O.C.T. compound for cryosectioning by the Histopathology core at UT Southwestern. Confocal imaging was performed in the Cell Biology and Imaging Core in the O'Brien Kidney Research Center, using a Zeiss LSM880 Airyscan laser scanning microscope equipped with Plan-Apochromat 10×/0.3 numerical aperture (NA), 20×/0.8 NA, 25×/0.8 NA and 63 × /1.40 NA oil immersion objectives (Zeiss). Fluorescence images were acquired using Zeiss ZEN black 2.3 software with a 20×/0.8 NA or 63×/1.40 NA objective and Zeiss immersion oil 518F was used for 63×/1.40 NA at constant RT. Acquired images were analyzed and regions of interest were further processed with Zeiss ZEN 2.6 (blue edition) software.

### Isolation of cardiac myocytes and fibroblasts

Mouse hearts were perfused and digested with collagenase using methods previously described (41). Ventricles were removed from digested hearts and mechanically dispersed in medium by trituration in a 50 ml conical flask. Dispersed cells were separated by size by centrifugation for 5 min at 50*g* in a swing bucket centrifuge. Upper 90% of the mixture that contained most of the nonmuscle cells was transferred to a 10 cm tissue culture dish. The lower 10% was washed once more and then myocyte cell pellet transferred to dishes with laminin-coated coverslips. Myocytes were allowed to attach for at least 3 h prior to fixation and incubation with antibodies for immunofluorescence measurements. Nonmuscle cells were allowed to attach for 3 h, then gently washed free of cells and debris with PBS prior to incubation in a humidified incubator at 37 °C with 5% CO_2_. After recovery overnight, adherent cells were trypsinized and replated onto a new dish and allowed to attach for 3 h and washed free of unattached cells. Adhesion-selected cells were >85% fibroblasts. Fibroblast-enriched cells were cultured for additional 1 to 3 days for immunoblotting and confocal microscopy.

### Myofibril dephosphorylation assay

Mouse cardiac myofibrils were prepared as previously reported with modifications (8). Mouse ventricles were dissected, homogenized in 30× weight adjusted volumes of heart extraction buffer (50 mM Mops pH 7.4, 2 mM EDTA, 2 mM EGTA, 150 mM NaCl, 1× Halt, 10 μM e64, 1 mM DTT, and 1% Triton X-100) with a ground glass homogenizer, incubated on ice for 30 min, and collected by centrifugation (1000×*g*, 5 min) in a microcentrifuge tube. For all subsequent wash steps, the homogenate pellet was hand homogenized with a plastic homogenizer directly in the microcentrifuge tube, and myofibrils were collected by low speed centrifugation (300×*g*, 1 min). Myofibrils washed 1× in heart extract buffer without Triton X-100 and then 2× times in 8× volume of myofibril assay buffer (50 mM Mops, 10 mM MgCl_2_, 2 mM EGTA, 25 mM NaCl, 10 μM E64, 1× Halt Protease inhibitor cocktail [Thermo], and 1 mM DTT). Myofibrils were resuspended in 8× volume of myofibril assay buffer and used immediately or resuspended in 4× volume of wash buffer with 50% glycerol and stored in −20 °C for up to 2 weeks.

Stored myofibrils in glycerol were washed in myofibril assay buffer to remove glycerol and then resuspended to the original 8× volume of myofibril assay buffer. Based on comparisons to published thick and thin filament protein stoichiometry (39) and starting wet tissue weight, the concentration of myosin RLC in washed myofibrils is approximately 20 μM. Myofibrillar RLC at 1 μM concentration in wash buffer was equilibrated with 100 nM skMLCK, 1.5 μM calmodulin, 100 μM blebbistatin, and 0.2 mM Na_2_ATP for 30 min on ice, prewarmed in a 30 °C waterbath for 5 min, and then phosphorylated for 30 min with the addition of 2.5 mM CaCl_2_ (0.5 mM free). Added reagents contributed <5% of total volume. Phosphatase reactions were initiated at the end of 30 min with the addition of 5 mM EGTA, then 40 μl aliquots removed and precipitated in equivalent volume of 20% TCA/20 mM DTT on ice at times indicated to stop all reactions. At end of assays, TCA-precipitated proteins were centrifuged at 300*g* for 2 min at 4 °C and then processed using same procedures as TCA-precipitated tissues. Myofibrillar protein precipitates were resolubilized by shaking vigorously (1400 rpm) for at least 2 h at RT with added urea crystals to maintain saturating conditions. Muscle myofibril proteins in an 8M urea buffer were processed for RLC phosphorylation measurements by urea/glycerol-PAGE as detailed previously.

### Statistical analyses

Data are expressed as mean ± standard error. Statistical evaluation was carried out in GraphPad Prism using an unpaired Student’s *t* test for 2 comparisons or paired *t* test for comparison of the same sample before and after treatment. One-way or Two-way ANOVA followed by multiple comparisons post-tests were used to determine significance. Specific tests used in comparisons are described in figure legends. Significance was accepted at a value of *p* < 0.05.

## Data availability

All data are contained in the article.

## Supporting information

This article contains supporting information (4 supplemental figures).

## Conflict of interest

The authors declare that they have no conflicts of interest with the contents of this article.
