# Supplementary material for: Myosin light chain phosphatase catalytic subunit dephosphorylates cardiac myosin via mechanisms dependent and independent of the MYPT regulatory subunits
Source: J Biol Chem. 2022 Jul 21;298(9):102296. doi: 10.1016/j.jbc.2022.102296 (PMC9418503; doi:10.1016/j.jbc.2022.102296)
Supplement: Supplemental Figures S1–S4 [file mmc1.docx]

Supplementary Figures.

Figure S1. Immunofluorescence of mouse heart sections from control WT and PP1cβ KO mice. Cryosections were co-immunostained with antibodies against PP1cβ (green) and smooth muscle α-actin. Large merge image scale bar indicates 20μm.

Figure S2. Comparison of smooth muscle α-actin immunofluorescence in adhesion-selected fibroblasts from control and PP1cβ KO mouse hearts. Scale bar: 100 μm in 10x images, 50 μm in 20x images.


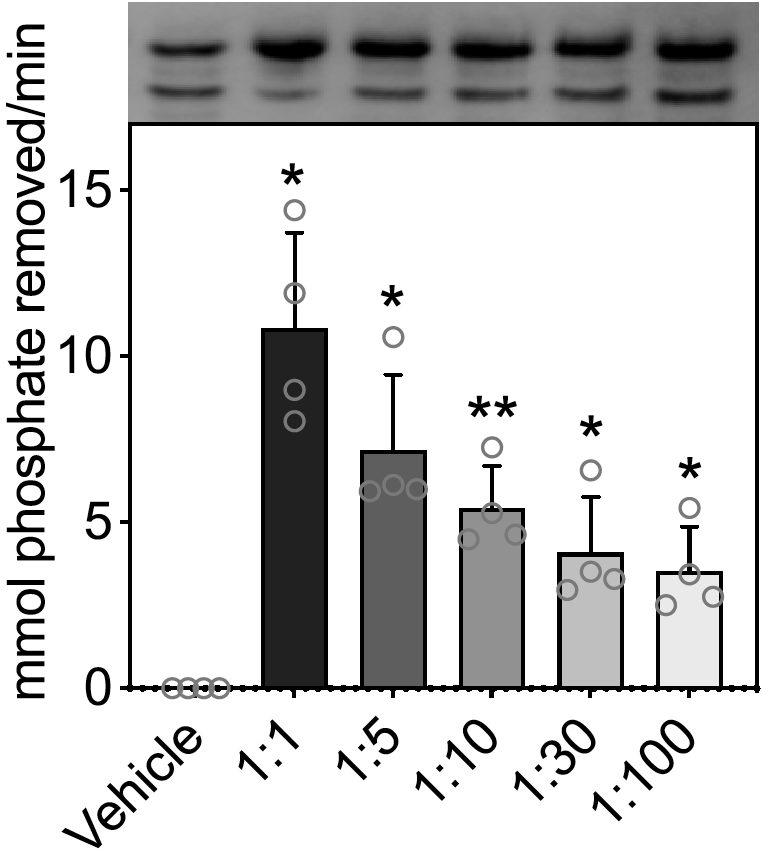


RLC

pRLC

Figure S3. Optimization of supernatant fraction dilution factor in myofibrillar dephosphorylation assay. Dephosphorylation of pre-phosphorylated myofibrillar myosin in 20 minutes by additions of supernatant fractions to myofibrils at indicated dilutions. *P<0.05, **P<0.01 compared to myosin bound MLCP activity in vehicle treated myofibrils, N=4.

Figure S4. Immunofluorescence images of mouse heart cryosections stained with the macrophage marker F4/80 and MYPT1. Upper panels show low magnification of mouse ventricular free wall transverse section; scale bar is 20 μm. F4/80 positive immunofluorescence and MYPT1 immunofluorescence are both increased. Enlarged region is indicated in merge panel. Lower set of enlarged images show increased intensity of MYPT1 in F4/80 positive cells, indicating MYPT1 protein is expressed in macrophages.
